# Starvation-induced long non-coding RNAs are significant for prognosis evaluation of bladder cancer

**DOI:** 10.18632/aging.204444

**Published:** 2022-12-20

**Authors:** Chunlin Zhang, Xuesong Bai, Xiang Peng, Wei Shi, Yang Li, Guo Chen, Haitao Yu, Zhenwei Feng, Yuanzhong Deng

**Affiliations:** 1 Department of Urology, The First Affiliated Hospital of Chongqing Medical University, Chongqing 400016, China; 2 Chongqing Key Laboratory of Molecular Oncology and Epigenetics, Chongqing 400016, China

**Keywords:** bladder cancer, starvation, lncRNA, prognosis

## Abstract

Background: Starving intratumoral microenvironment prominently alters genic profiles including long non-coding RNAs (lncRNAs), which further regulate bladder cancer (BCa) malignant biological properties, such as invasion and migration.

Methods: Transcriptome RNA-sequencing data of 414 BCa tumor tissues and 19 normal tissues were obtained from TCGA database and paired samples of 132 BCa patients. A chain of *in vitro* validations such as qPCR, migration and invasion assays were performed to reveal the clinical relevance of AC011472.4 and AL157895.1.

Results: A total of 11 lncRNAs were identified as starvation-related lncRNAs, of which AC011472.4 and AL157895.1 were relevant to overall survival of BCa patients. Besides, a starvation-related risk score model was established based on the levels of AC011472.4 and AL157895.1. BCa patients with higher levels of AL157895.1 were divided into the high-risk group and usually obtained higher mortality rate, but AC011472.4 was contrary. AL157895.1 expressed highly in BCa cell lines and tumour tissues, especially in patients with the advanced grade, stage and T-stage, while AC011472.4 showed the reversed result. Moreover, increased level of AL157895.1 was remarkably correlated to T-stage, muscle invasion status and distant metastasis. SiRNAs-mediated silence of AC011472.4 and AL157895.1 respectively increased and diminished invasion and migration properties of BCa cells.

Conclusions: In this study, we highlight the significant roles of AC011472.4 and AL157895.1 on evaluating prognoses of BCa patients and validate their correlation with various clinical parameters. These findings provide an appropriate risk score model for BCa clinical decision making.

## INTRODUCTION

Bladder cancer (BCa) is the most common genitourinary malignancy which is characterized by high recurrence rate and rapid progression [1]. There are more than 500,000 new cases worldwide each year, ranking 11^th^ among all malignant tumors, 4^th^ and 19^th^ among male and female malignant tumors respectively [2, 3]. Near 25% of patients diagnosed with BCa present with muscle-invasion or distant metastasis, and these patients typically suffer with the poor prognoses and worse quality of life [4]. Surgery combined with chemoradiotherapy and targeted immunotherapy are the mainstream options for BCa [5]. However, most treatment plans are troubled by a high recurrence rate, low response rate and drug resistance, resulting in limited benefits for patients, especially for local advanced BCa and metastatic BCa [5–7]. After radical cystectomy, about 40% of patients suffered with local recurrence or distant metastasis, and the 5-year overall survival was only 14-15 months [4, 8].

In addition, BCa, as a highly heterogeneous disease, exhibits various tumor characteristics because of different genomic maps among patients, causing distinct therapeutic responses. Along with the continuous improvements in genomics and proteomics, cancer precise strategies have entered a stage of rapid leaps in development [9, 10]. In the past decades, precise diagnosis and therapy based on tumor-specific biomarkers including genes and proteins have been substantially developed and gradually applied to clinical management [11]. For instance, Kallikrein-related peptidase 6 was over-expressed in bladder tumour tissues and was validated to be closely associated with the BCa progression and poor prognosis [12]. HSP90B1 remodeled BCa’s immunological milieu by regulating endoplasmic reticulum stress signaling pathway and PD1 expression in immune clusters, thus HSP90B1 was identified as an optional therapeutic target and promising prognostic biomarker for BCa immunotherapy [13].

The rapid growth of tumor cells results in malformation of vascular distribution within bladder tumor, further evoking nutrient-deprived conditions [14]. Starving environment remarkably altered cellular profiles at the levels of gene and protein, and regulated activities of different subpopulations. Long-term serum starvation induced constitutive ErbB/MAPK activity and promoted BCa progression [15]. Nutrient-deprived environment significantly enhanced the invasion and migration of BCa cells by activating TGF-β1/Smad3-mediated epithelial-mesenchymal transition [16]. Starvation-induced autophagy promoted BCa to secrete small extracellular vesicles and provoked angiogenesis by reprogramming HBP-related glucose metabolism [17]. Therefore, identifying remarkable gene alternations under starvation conditions can help to find more attractive and viable targets.

Long non-coding RNAs (lncRNAs) are a group of single-stranded nucleotide sequences, which are non-coding but play extremely prominent role on various biological processes, including tumorigenesis, metastasis and malignant progression [18–23]. A huge number of studies have suggested that lncRNAs are over-expressed in bladder tumour tissues and play nonnegligible roles in early diagnosis and prognostic evaluation [24]. The levels of lncRNAs (MKLN1-AS, TALAM1, TTN-AS1 and UCA1) are significantly increased in urinary exosome of BCa patients and a panel of these lncRNAs is capable of classifying BCa patients versus non-BCa population. Ferroptosis-related lncRNA-AC006160.1, are down-regulated in BCa cells, and obviously attenuated proliferation and invasion, thus serves as a protective biomarker for assessing BCa prognosis [25]. However, the effect of starvation-mediated alteration of lncRNA on BCa diagnosis and prognosis evaluation remain poorly understood, and need further discovery and attention.

In this study, we aim to establish a novel BCa prognosis risk model based on starvation-related lncRNAs (SRLNRs) for long-term prognostic evaluation. In addition, the clinical significance of the starvation-related risk score model is validated in a single-center-based cohort.

## RESULTS

### Establishing the starvation-related risk score model by survival-related SRLNRs

**Figure 1:**
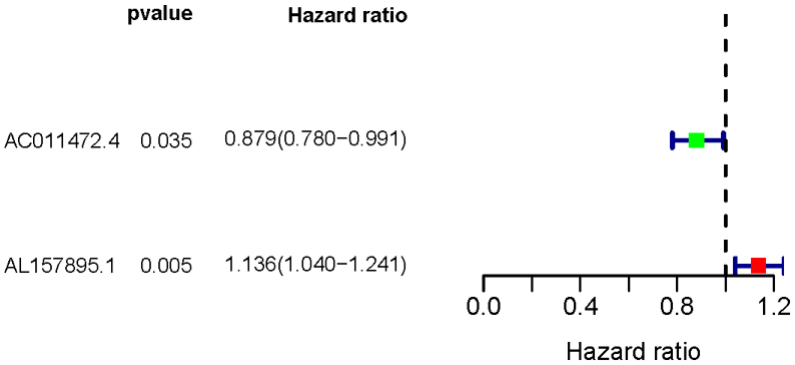
**The forest plot of sSRLNRs.** The forest map showed the hazard ratios of AC011472.4 and AL157895.1. The green color represents the positive correlation with BCa patients’ prognoses, while the red color represents the opposite relation.

**Figure 2:**
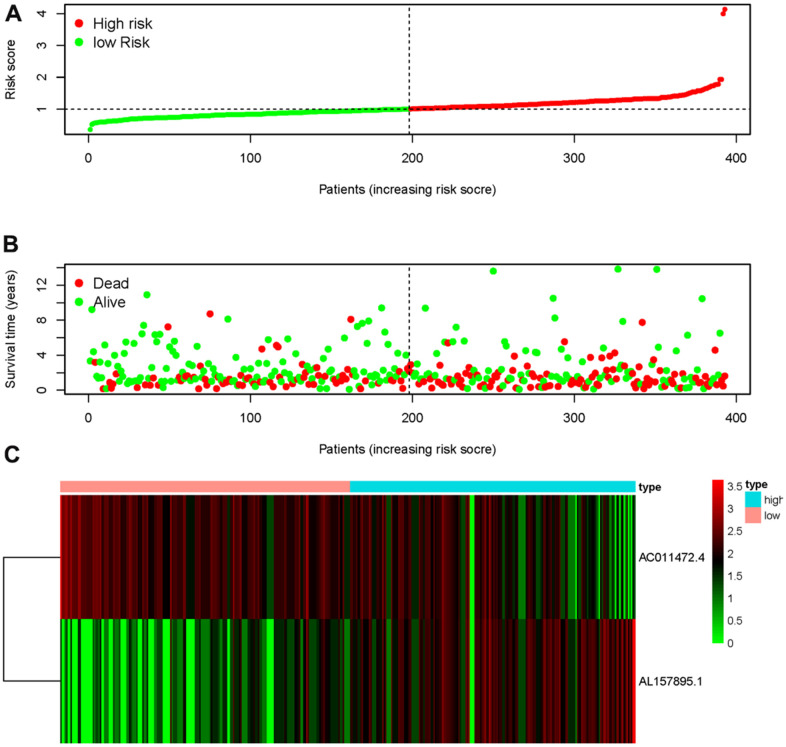
**Starvation-related risk score model.** The expression level of AL157895.1 (**A**) and AC011472.4 (**B**) in tumor tissues and adjacent normal bladder tissues. The heatmap of expression levels of AC011472.4 and AL157895.1 in the SRRSM (**C**).

**Figure 3:**
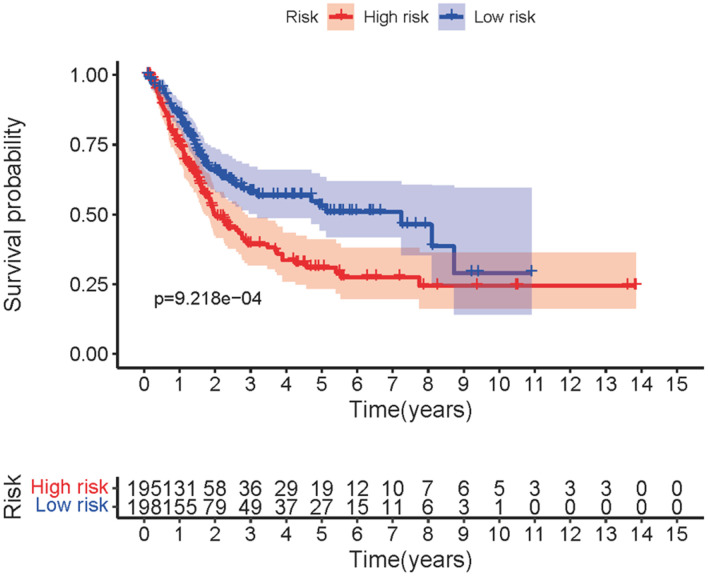
**Survival curve of SRRSM.** Kaplan-Meier survival curve of the high-risk group and the low-risk group in the SRRSM.

Transcriptome RNA-sequencing data and clinical data of BCa patients were downloaded from TCGA database. Following that, we screened 353 SRGs in the M16522 and M41835 of Molecular Signatures Database and analyzed the correlation between lncRNAs and 352 SRGs by Pearson correlation analysis. 11 lncRNAs were associated with starvation (|r|>0.7 and *P*<0.01). To detect the relationship between SRLNRs and the prognosis of BCa patient, we then performed the univariate COX regression analysis. As illustrated in the forest map, AC011472.4 was regarded as a protective factor but AL157895.1 is a deleterious factor (Figure 1). We then used multivariate COX regression analysis to establish the SRRSM. According to the median risk score, BCa patients were divided into the high-risk group and the low-risk group (Figure 2A). With the increasing risk score, the mortality rate of BCa patient and the expression level of AL157895.1 were constantly increased, while the expression of AC011472.4 decreased (Figure 2B). The heatmap showed that AL157895.1 was highly expressed in the high-risk group while the expression of AC011472.4 was decreased in the high-risk group (Figure 2C). In addition, Kaplan-Meier survival curve showed that patients in the high-risk group obtained poorer prognoses (Figure 3).

### Clinical relevance of sSRLNRs

**Figure 4:**
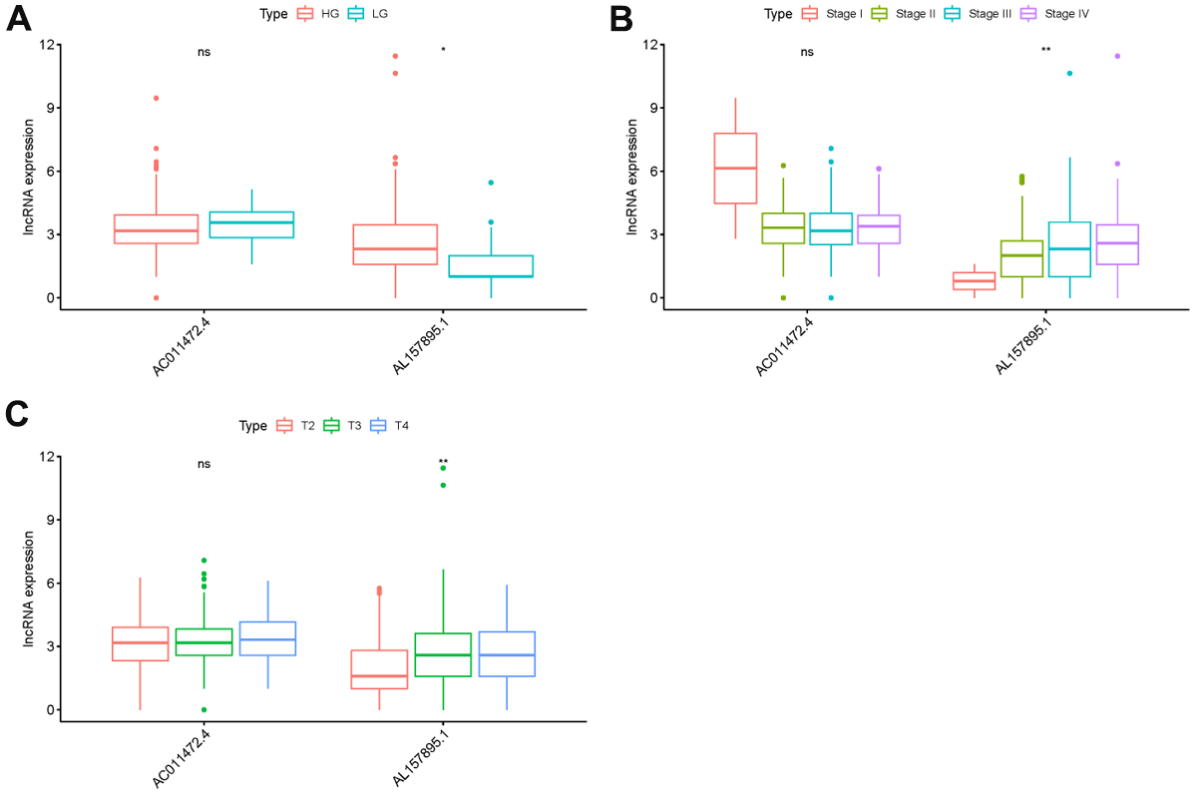
**Receiver operating characteristic (ROC) curves.** The area under curves (AUCs) of the 1-,3- and 5-year SRRSM. The 1-,3-,5-year AUCs’ risk scores were 0.632 (**A**), 0655 (**B**) and 0.705 (**C**) respectively.

**Figure 5:**
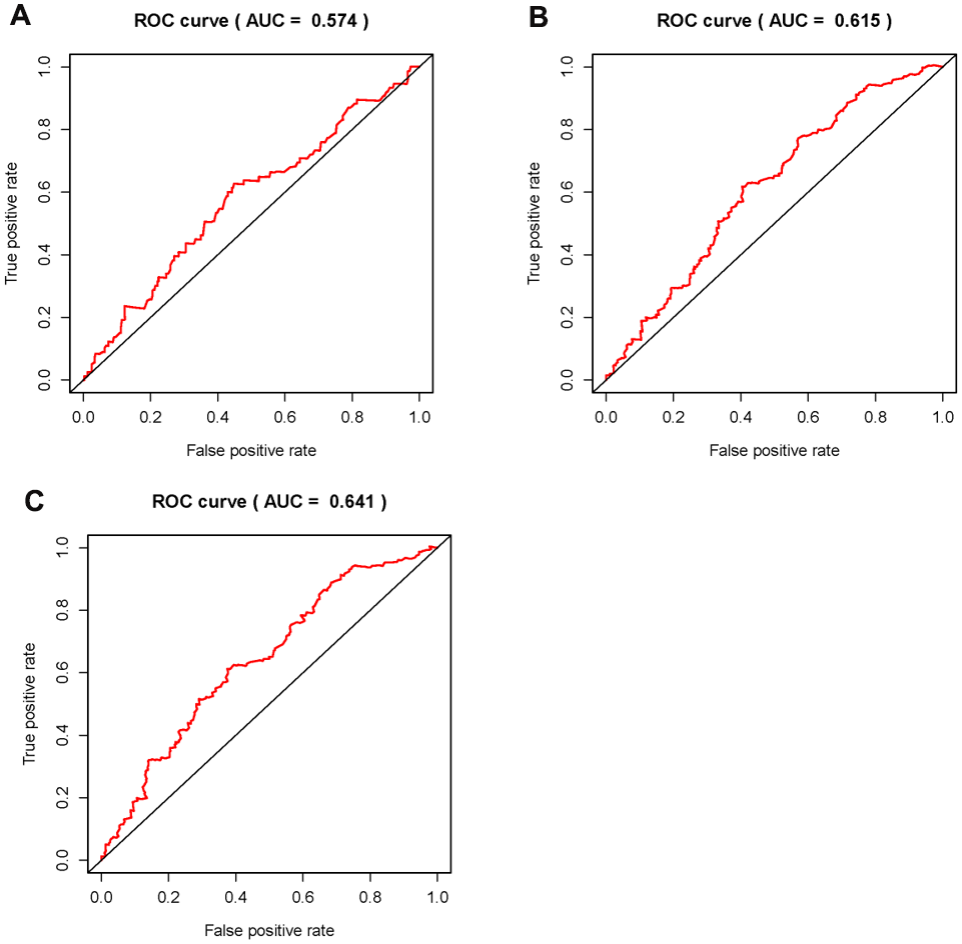
**Clinical correlation analysis of AC011472.4 and AL157895.1.** The expression level of AL157895.1 was increased in the more advanced grade (**A**), stage (**B**) and T-stage (**C**).

**Figure 6:**
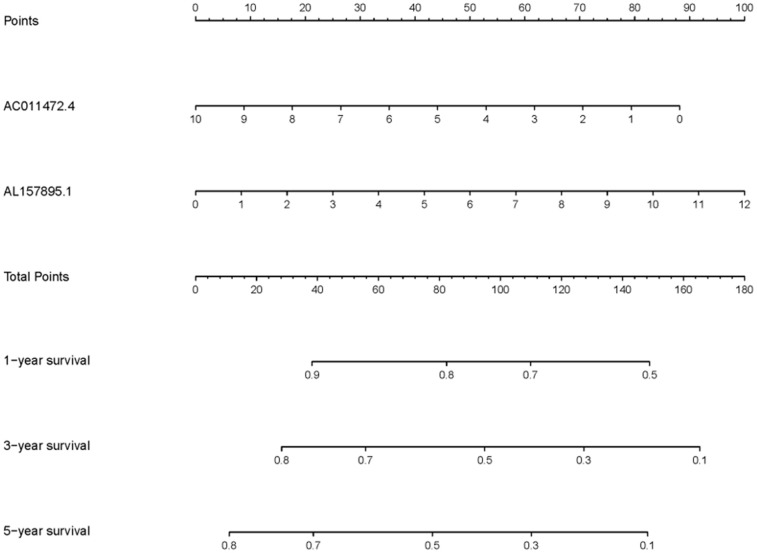
**Clinical application of SRRSM.** The nomogram of SRRSM could predict the 1-, 3- and 5-year survival probabilities of BCa patients.

In order to explore the relevance of sSRLNRs and clinicopathologic features of BCa patients, such as grade, stage, T-stage, N-stage and M-stage, we applied ggpubr package and found that AL157895.1 was highly expressed in BCa patients with the advanced grade, stage and T-stage (Figure 4A–4C). Following that, we performed multivariate analysis to detect the underlying independent risk factor of BCa patients, and the results showed that only risk score could serve as an independent risk factor of BCa patients (Table 1). In order to detect the accuracy of SRRSM, we calculated the AUCs for ROC curves of SRRSM and clinical characters, and found the AUCs of 1-,3- and 5-year risk score were 0.574, 0.615 and 0.641 respectively (Figure 5A–5C). These results suggested that SRRSM was the most accurate independent risk factor of BCa patients. Then, we normalized the points of SRRSM ranging from 0 to 100, and calculated the 1-year, 3-year and 5-year survival probabilities by drawing expression levels of sSRLNRs line between the total points axis and each prognosis axis (Figure 6). These results illustrated that the SRRSM could be served as a risk factor to predict the prognoses of BCa patients.

**Table 1:** Multivariate COX analysis of BCa patients.

**Variables**	**Multivariate analysis**			
	**HR**	**HR 95% low**	**HR 95% high**	**P value**
**Age**	1.018120	0.991100	1.045876	0.190678
**Gender**	0.615066	0.356895	1.059993	0.080090
**Grade**	0.706623	0.090811	5.498390	0.740093
**Stage**	1.322635	0.654847	2.671405	0.435613
**T-stage**	1.264981	0.767972	2.083641	0.355930
**M-stage**	1.436841	0.492539	4.191568	0.506997
**N-stage**	1.146347	0.692501	1.897630	0.595342
**Risk score**	3.786814	1.540670	9.307610	0.003708

Note: HR, Hazard Ratio.

### The underlying mechanism of SRRSM

**Figure 7:**
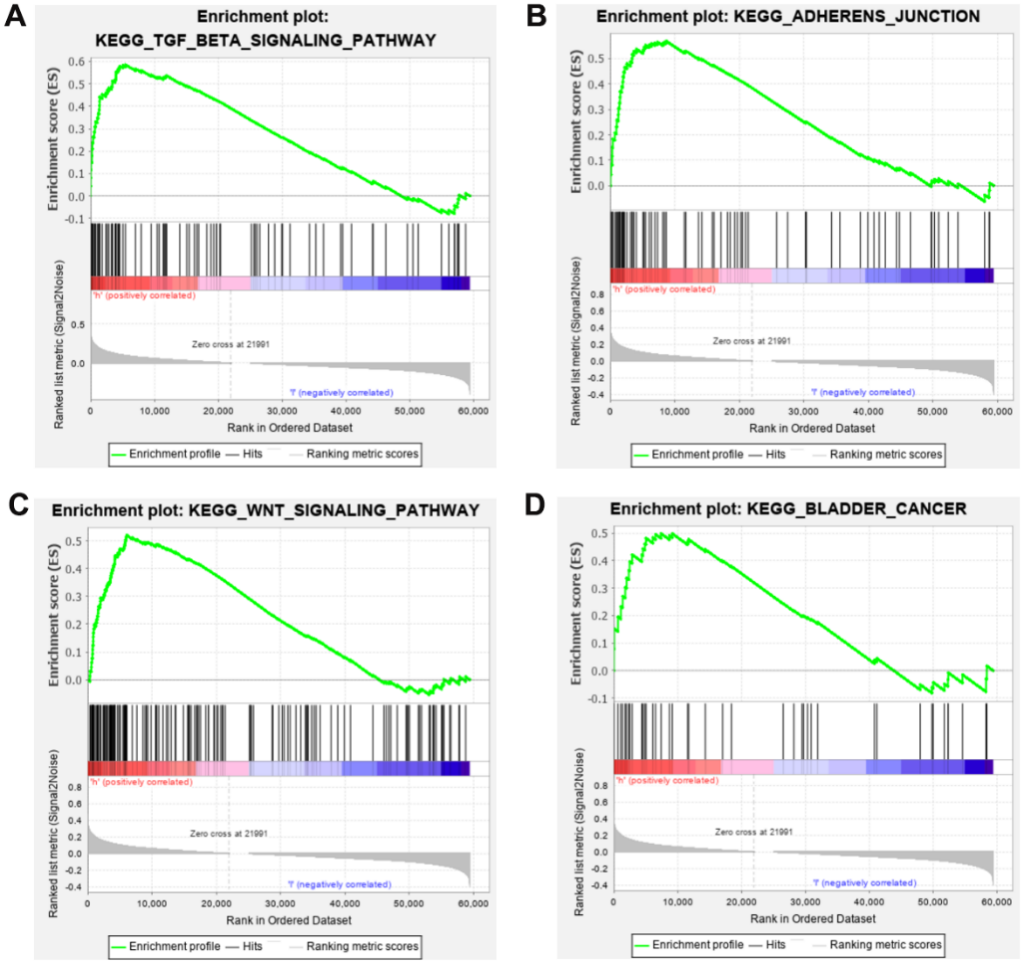
**Gene set enrichment analysis (GSEA) of SRRSM.** GESA illustrated that the high-risk group patients were significantly enriched in TGF-beta (**A**), adherens junction (**B**), WNT (**C**) and bladder cancer pathway (**D**).

In order to detect the underlying mechanisms of SRRSM, the Kyoto Encyclopedia of Genes and Genomes (KEGG) pathway analysis of GSEA were employed. We found that the high-risk group was closely related to the TGF-beta, ADHERENS, WNT and BLADDER CANCER signaling pathway (Figure 7A–7C). These results prompt us to further detect associations of SRLNRs with invasion and migration in subsequent studies.

### AC011472.4 and AL157895.1 obtain prominent clinical relevance and significance

**Figure 8:**
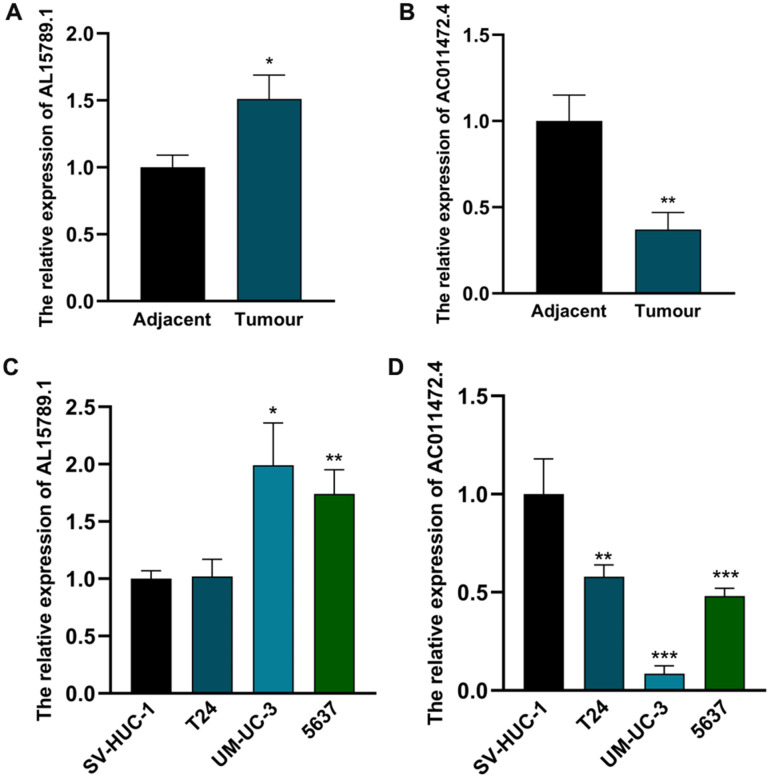
**The expression levels of AC011472.4 and AL157895.1 in cell lines and clinical samples.** The expression levels of AL157895.1 (**A**) and AC011472.4 (**B**) in tumor tissues and adjacent normal bladder tissues. The expression levels of AL157895.1 (**C**) and AC011472.4 (**D**) in BCa cell lines and SV-HUC-1 cell line.

Next, we detected the expression levels of AC011472.4 and AL157895.1 in various bladder urothelial cell lines and tissues. As illustrated in Figure 8A, 8B, the expression level of AL157895.1 in bladder tumour tissue was prominently higher than that in adjacent normal tissue, while AC011472.4 expressed inversely. Consistently, we detected significantly higher expression of AL157895.1 in two BCa cell lines (UM-UC-3 and 5637) compared to normal urothelial cell line (SV-HUC-1), but AC011472.4 expressed reductively in BCa cell lines (Figure 8C, 8D).

Additionally, based on the expression level of AL157895.1 in tumour tissue (cut-off value: 0.3250), 132 BCa patients were separated into the high-expression group (n = 66) and the low-expression group (n = 66). We found that AL157895.1 showed remarkable correlation to T stage. muscle invasion status and distant metastasis status, while different from the result of TCGA, we didn’t detect the prominent associations with grade and stage (Table 2).

**Table 2:** The relations between AL157895.1 and clinicopathologic features of BCa patients.

**Parameter**	**N**	**Expression of AL157895.1**		***P* value**
		**Low**	**High**	
**Gender**				0.827
**Male**	106	54	52	
**Female**	26	12	14	
**Age (year)**				0.137
**<70**	89	40	49	
**≥70**	43	26	17	
**T stage**				< 0.001
**1-2**	97	59	38	
**3-4**	35	7	28	
**Muscle invasion status**				0.023
**Negative**	41	27	14	
**Positive**	91	39	52	
**Lymph node status**				1.000
**Negative**	127	64	63	
**Positive**	5	2	3	
**Distant metastasis status**				0.001
**Negative**	115	64	51	
**Positive**	17	2	15	

### AC011472.4 and AL157895.1 were starvation-related and significantly affected the invasion and migration abilities of BCa cell

**Figure 9:**
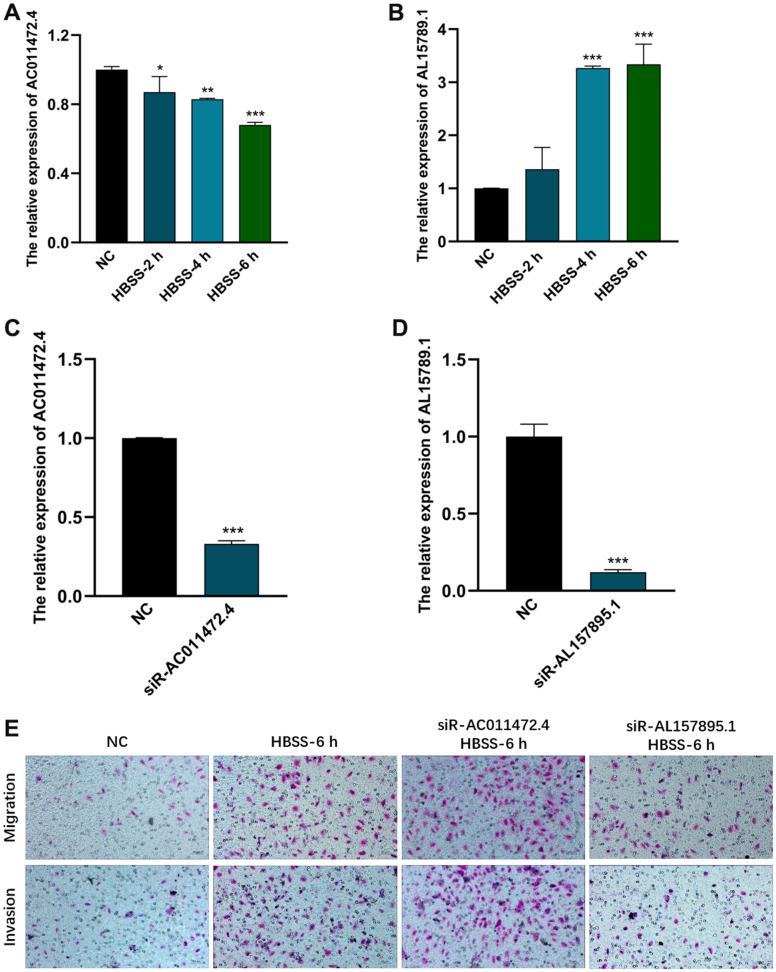
**The effects of AC011472.4 and AL157895.1 on invasion and migration under starvation condition.** The expression level of AC011472.4 (**A**) decreased under starvation condition, but AL157895.1 (**B**) increased. The knock-down efficiency of AC011472.4 (**C**) and AL157895.1 (**D**). Transwell assays showed the migration and invasion abilities of BCa cells following different treatments (**E**).

In order to explore the roles of AC011472.4 and AL157895.1 in the starvation tumor microenvironment. We first established HBSS-induced starvation model *in vitro*, and examined the expression levels of AC011472.4 and AL157895.1under the starving condition. The results showed that the expression of AL157895.1 was significantly up-regulated in the starvation tumor microenvironment, while AC011472.4 expressed decreasingly in a time-dependent manner (Figure 9A, 9B). To further validate whether AC011472.4 and AL157895.1 affect the metastatic potency of BCa, the expression of AC011472.4 and AL157895.1 was first silenced by siRNAs in UMUC-3 cells (Figure 9C, 9D). Next, we performed the invasion and migration assays and found that starvation-induced BCa cells acquired higher abilities of invasion and migration, which were prominently inhibited in siR-AL157895.1-treated cells, but further elevated in siR-AC011472.4-treated cells (Figure 9E). Together with above results, we can conclude that, in the starvation tumor microenvironment, AC011472.4 and AL157895.1 play crucial roles in BCa metastasis.

## DISCUSSION

The invasiveness and migration of tumour cells has long severely threatened survival of BCa patients [26]. About 30% of BCa patients present with muscle-invasive or metastatic status, and approximately half of muscle-invasive BCa patients relapse after radical cystectomy, most of which accompanied with distant metastasis [27]. Series of evidence has demonstrated the crucial roles of lncRNAs on regulating BCa metastasis [24, 28]. Increasing expression of LINC01929 in BCa cells upregulated ADAMTS12 by competitively adsorbing miR-6875-5p and then promoted invasion, progression, and metastasis of advanced BCa [29]. However, LINC00478 illustrated anti-cancer effects by targeting lysine-specific demethylase-1 and suppressing the expression of matrix metalloprotein 9 [30]. Exosomal LINC00960 and LINC02470 secreted by high-grade BCa cells increased the malignant potency of low-grade BCa cells and induced epithelial-mesenchymal transition by activating β-catenin, Notch and Smad2/3 pathways [31]. In the present study, we first demonstrated that AL157895.1 was closely correlated to the increase of invasion and migration, but AC011472.4 showed completely inverse effect. In addition, AC011472.4 has ever been reported that promoted colorectal cancer progression by regulating Toll-like receptor [32]. AL157895.1 was identified as a kind of N7-methylguanosine-related lncRNA which inhibited immune infiltration and checkpoint expression, thus predicted poor effect on immunotherapy.

Accumulating evidence has unraveled that stressful tumour environment significantly altered tumour cell behavior by genic and epigenetic modulation. LncRNA NEAT1 was over-expressed in multiple myeloma cells exposed in nutrient-deprived environment and provoked higher aggression by inducing two fundamental kinases (ATM and DNA-PKcs) and their targets (pRPA32 and pCHK2) [33]. On contrary, lncRNA TINCR was depleted under the normal oxygen condition, so as to mediate global translational reprogramming and increased proteins synthesis of ATF4 and other integrated stress response, leading to melanoma metastasis [34]. Furthermore, starvation-induced inhibition of MAPK signaling pathway and cell cycle arrest increased the level of LINC00483, which reversed inhibited the epithelial to mesenchymal transition, provoking a modest decrease of cell migration rate [35]. In the study, we, for the first time, revealed that starving tumor microenvironment prominently increased AL157895.1 level and attenuated AC011472.4 level in a time-dependent manner. Moreover, these starvation-related lncRNAs both significantly regulated BCa invasion and migration.

Although these findings unravel the potential of SRRSM for prognosis assessment for BCa patients and validate the close associations of starvation-related lncRNAs (AC011472.4 and AL157895.1) with various clinicopathologic features, some limitations are still needed to be overcome. Extracellular vesicles have been increasingly demonstrated to mediate transcellular transport of lncRNA in tumour microenvironment, therefore, whether AC011472.4 and AL157895.1 are also loaded in BCa cells-secreted extracellular vesicles and affect behaviors of other subcellular populations remain unknown. In addition, underlying mechanisms by which nutrient-deprived environment regulates starvation-related lncRNAs levels and AC011472.4 and AL157895.1 modulate BCa invasion and migration, are also needed to be further investigated. In future studies, more *in vivo* and *in vitro* models should be established to elucidate detailed molecular mechanisms underlying lncRNAs-mediated BCa progression, besides, multiple omics assays are essential to reveal secrets of starving intratumoral microenvironment from the deeper and wider perspectives.

## CONCLUSIONS

In the present study, we illuminate the promising effects of sSRLNRs in prognosis evaluation for BCa patients and ascertained the clinical significance and metastasis relevance, after the validation in BCa tissues and different BCa cell lines. AL157895.1 with higher expression in tumour tissue was validated to further promote BCa metastasis under starving condition, while AC011472.4 showed reversed results. These results not only establish a link between sSRLNRs and BCa invasion and migration, and also develop an appropriate SRRSM for BCa metastatic risk assessment.

## MATERIALS AND METHODS

### Clinical samples

One hundred and thirty-two bladder tumor tissues and adjacent normal tissues were collected from patients who underwent tumor excision in the First Affiliated Hospital of Chongqing Medical University between March 2019 and June 2022 were collected (Table 2). The collected tissues were immediately frozen in liquid nitrogen until RNA extraction.

### Cell culture and treatment

SV-HUC-1 and BCa cell lines (T24, UM-UC-3 and 5637) were purchased from the American Type Culture Collection (Manassas, Virginia, USA). Cells were cultured in DMEM/F12 (SV-HUC-1), DMEM (UM-UC-3) and RPMI-1640 (5637 and T24) basal medium (Gibco, Gaithersburg, MD, USA) and supplemented with 10% fetal bovine serum (Biological Industries, IL), 100 U/ml penicillin and 0.1 mg/ml streptomycin (Beyotime, Beijing, China). Cells were incubated at 37° C in 5% CO_2_ incubator and the medium was changed every 2-4 days. Starvation-induced UMUC3 cells were treated with Hank’s solution (Boster Biotechnology, China) for six hours and were then recovered in complete DMEM medium.

### Cell transfection

The siRNAs of AC011472.4 and AL157895.1 were transfected to silence the expression of AC011472.4 and AL157895.1. The sequences used were: siR-AC011472.4 (sense:5′-AGAUGAGUUGGACAUCCUACU-3′, antisense: 5′-UAGGAUGUCCAACUCAUCUUU-3′); siR-AL157895.1 (sense: 5′-GAAGGAAGAUGAUAUUUAAGA-3′, antisense: 5′-UUAAAUAUCAUCUUCCUUCUU-3′). For transient transfection, UMUC-3 cells were cultured into 6-cm dish (5×10^5^ cells). When cells grew to 40 % of surface, cells were transfected with 10μl siRNAs (20uM) using 5μl Lipofectamine 3000 (Invitrogen, USA) for 48h according to the manufacturer and used RT-qPCR to verify the efficiencies of siRNAs.

### Real-time quantitative PCR

Trizol (Abclonal) was used to extract total RNA from cell lines and clinical tissues. 1μg RNA was displayed to reverse transcribed cDNA by cDNA Synthesis Kit (Abclonal). The quantitative PCR (qPCR) was run on ABI 7500 real-time PCR system (Applied Biosystems) by SYBR-Green method (Abclonal). 2^−ΔΔCt^ method was used to calculate the values of Ct. The lncRNA values were normalized to the expression levels of β-actin. The expressions of lncRNAs were relative to the fold change of their controls which were defined as 1. The primer sequences of lncRNAs and β-actin were shown in Table 3. Three assays were conducted per cDNA sample.

**Table 3:** The sequences of AC005625.1, AC008760.1 and β-actin.

**AC011472.4**	F primer (5’-3’)	ctgtggctataccttagaccctcagtc
	R primer (5’-3’)	acacacacagacaccctggg
**AL157895.1**	F primer (5’-3’)	tggtcaaagcctgagcatacaacc
	R primer (5’-3’)	aagtatcgttattctttctacaaacattcagttgattcttac
**β-actin**	F primer (5’-3’)	ggctattctcgcagctcacc
	R primer (5’-3’)	gtgtaacgcaactaagtcatagtccgc

Note: F primer, forward primer; R primer, reverse primer.

### Transwell assay

For the migration assay, 600μl DMEM medium containing 10% FBS was added into the lower chamber, and 4×10^5^ UMUC3 single-cell suspension was seeded in the upper chamber (Corning, USA). For the invasion assay, Matrigel (1:9 dilution with the DMEM basal medium) was added into the upper chamber. Two hours later, 6×10^5^ UMUC3 cells in 100μl DMEM basal medium were added into the chamber with diluted Matrigel and put in the lower chamber containing 600ul complete DMEM medium. After 24 and 48 hours of culturing for the migration test and invasion test, additionally, the inserts were washed with PBS and fixed with 4% paraformaldehyde for 20 minutes. Next, inserts were stained with 0.1% crystal violet solution for 20 minutes. The 5 fields (200X) of the insert were photographed by light microscopy.

### BCa transcriptome data of TCGA preprocessing

Transcriptome RNA-sequencing data of 414 BCa tumor tissues and 19 normal tissues were downloaded and extracted from The Cancer Genome Atlas (TCGA) data portal (https://portal.gdc.cancer.gov/). We excluded patients whose OS ≤ 30 days from this study because they might die of unpredictable factors such as hemorrhage and infection. Raw data of BCa patients were collected for further analysis. Transcriptome RNA-sequencing results and clinical data of BCa patients were combined into a matrix file by a merge script in the Perl language (http://www.perl.org/).

### Acquiring the survival-related SRLNRs (sSRLNRs)

Starvation-related genes (SRGs) were downloaded from The Molecular Signatures Database v4.0 (M16522 and M41835, http://www.broadinstitute.org/gsea/msigdb/index.jsp). We conducted the Pearson correlation analysis to detect the correlation between SRGs and the lncRNA expression levels of BCa patients. A standard of |r|>0.7 and *P*<0.01 was used to screen the SRLNRs. Furthermore, we screened the sSRLNRs by univariate COX analysis and survival packages of R software (*P*<0.01). Hazard ratio (HR) was used to divide sSRLNRs into deleterious and protective portions.

### Starvation-related risk score model (SRRSM)

Through multivariate COX regression analysis, we established the SRRSM based on the selected sSRLNRs. The risk score of BCa patients was calculated by the expression levels of sSRLNRs times the Cox regression coefficients. The formula was as followed, [Expression levels of AC011472.4 * (-0.150025465)] + [Expression levels of AL157895.1 * (0.141591127)]. BCa patients were separated into the high-risk group and the low-risk group according to the median score.

### Bioinformatics analysis

We evaluated the survival rate of patients in SRRSM by survival package. The receiver operating characteristic (ROC) curves and areas under curves (AUCs) were generated and calculated by survival ROC package to assess the accuracy of the SRRSM. Gene set enrichment analysis (GSEA) was used to detect the underlying pathways of SRRSM. We performed multivariate Cox regression analyses to verify the independent prognostic factor of BCa patients. Nomogram was used to predict the survival rate of BCa patients by the rms package.

### Statistical analysis

Statistical analysis was conducted by SPSS 21.0 software (SPSS Inc, Chicago, IL) and GraphPad Prism8 (GraphPad Software Inc, La Jolla, CA). Data were expressed as means ± SD. The correlations between sSRLNR expression and clinicopathological features were evaluated utilizing Fisher’s exact probability method. Student T-test, ANOVA and post-hoc test were utilized for difference comparison of two or more groups. *P*<0.05 was considered a significant statistical difference.

### Availability of data and materials

Authors can provide all of datasets analyzed during the study on reasonable request.
